# Structural Design of High-Coercivity Nd-Ce-Fe-B Magnets with Easy Axis Perpendicular Orientation and High-Abundance Ce Content Based on Micromagnetic Simulations

**DOI:** 10.3390/nano15171358

**Published:** 2025-09-03

**Authors:** Qian Zhao, Ying Yu, Chenlin Tang, Qingkang Hu, Suo Bai, Puyu Wang, Zhubai Li, Guoping Zhao

**Affiliations:** 1 School of Science, Inner Mongolia University of Science and Technology, Baotou 014010, China; 2 College of Physical Science and Technology, Heilongjiang University, Harbin 150080, China; 3 Rare Earth Industry Research Institute, Inner Mongolia University of Science and Technology, Baotou 014010, China; 4 School of Physics and Electronic Engineering, Sichuan Normal University, Chengdu 610101, China

**Keywords:** Nd-Ce-Fe-B, coercivity, main phase distribution, micromagnetics

## Abstract

In recent years, replacing the scarce and expensive rare earth element Nd with the more abundant and lower cost Ce in the production of Nd-Ce-Fe-B permanent magnets has become a focus of both industrial and academic research. A critical challenge is how to design the crystal structure of Nd-Ce-Fe-B magnets to compensate for the decline in magnetic performance caused by the Ce substitution. In this study, based on micromagnetic theory, Nd-Ce-Fe-B magnets with perpendicularly oriented easy axes—in which the two main phases, Nd_2_Fe_14_B and Ce_2_Fe_14_B, have a volume ratio of 1:1 but different spatial arrangements—are modeled and simulated using the MuMax3.11 software. The model is either cubic or spherical. The results from the demagnetization curve analysis indicate that the coercivity mechanism of all magnets is pinning. When the magnet volume is constant but the phase distribution differs, the Nd_2_Fe_14_B/Ce_2_Fe_14_B structure exhibits a higher coercivity and maximum energy product than the Ce_2_Fe_14_B/Nd_2_Fe_14_B structure. Furthermore, for both structural models with the same phase distribution, the coercivity and the maximum energy product decrease with the increasing volume of the main phase. Notably, the coercivity is similar when the magnet volume is very small and stabilizes after reaching a certain threshold. This qualitative conclusion was also observed in Nd-Dy-Fe-B magnets with the same structure and equal volume ratio of the two main phases. This general finding indicates that, in biphasic magnets with equal phase volumes, the phase with the larger anisotropy field located at the grain periphery can achieve a higher coercivity and maximum magnetic energy product. The analysis of the angular distribution reveals that the number of magnetic domains remains fixed at six in the Nd_2_Fe_14_B/Ce_2_Fe_14_B structure and two in the Ce_2_Fe_14_B/Nd_2_Fe_14_B structure. The in-plane magnetic moment analysis of the Ce_2_Fe_14_B/Nd_2_Fe_14_B magnet shows that the magnetic moments at the edges of the Ce_2_Fe_14_B begin to deflect first. Even at the pinning stage, the magnetic moments within the Nd_2_Fe_14_B remain unrotated. Nevertheless, the surface magnetic moments of Ce_2_Fe_14_B, through exchange coupling, drive the deflection of the interfacial and interior moments, completing the entire demagnetization process. These computational results provide theoretical guidance for related experimental studies and industrial applications.

## 1. Introduction

Since Kneller and Hawig [1] proposed the concept of exchange-coupled magnetic materials in 1991, both theoretical and experimental studies on exchange-coupled magnets have remained a research hotspot [2,3,4,5,6,7,8]. Nd_2_Fe_14_B is widely recognized as the best permanent magnet to date due to its high spontaneous magnetization and large crystalline anisotropy, which result in the highest measured energy product. Therefore, Nd_2_Fe_14_B has been selected as one of the most effective exchange-coupled materials. However, with the extensive consumption of heavy rare earth Nd, its crustal abundance has gradually declined, leading to a significant increase in cost. In contrast, Ce, a light rare earth element with a high crustal abundance and a relatively low cost, has attracted considerable attention in both industry and academia. Ce_2_Fe_14_B, although possessing a lower magnetic performance than Nd_2_Fe_14_B, is suitable for applications in medium- and low-grade permanent magnets. Consequently, Nd_2_Fe_14_B/Ce_2_Fe_14_B exchange-coupled dual-main-phase Nd-Ce-Fe-B magnets, whose magnetic properties lie between Nd_2_Fe_14_B and Ce_2_Fe_14_B, have become promising candidates for applications in devices such as variable magnetic force motors [9]. At present, experimental studies on Nd-Ce-Fe-B rare earth permanent magnets are relatively abundant [10,11,12,13,14,15,16,17,18], whereas theoretical investigations remain scarce [19,20,21]. To date, no systematic studies have been reported on the coercivity mechanism and energy product of Nd-Ce-Fe-B magnets using finite-difference micromagnetic simulations with MuMax3.

Considering that, in experiments or industrial production, the atomic fractions of the two main phases, Nd_2_Fe_14_B and Ce_2_Fe_14_B, can each reach up to 50%, and that the actual grain morphology is closer to a polyhedral structure between idealized cubes and spheres, this work employs the micromagnetic simulation software MuMax3 to design and model a high-coercivity Nd-Ce-Fe-B magnet with an easy axis perpendicular orientation and enriched Ce content. Simulations are carried out for cubic and spherical geometries with equal atomic fractions (50% each) of Nd_2_Fe_14_B and Ce_2_Fe_14_B. Magnetic hysteresis loops are calculated to obtain key physical quantities, such as the coercivity and maximum energy product. Furthermore, the in-plane magnetization and angular distributions are analyzed to investigate the coercivity mechanism from a microscopic perspective. This study provides theoretical guidance for experimental research and the industrial production of high-coercivity Nd-Ce-Fe-B magnets enriched with abundant rare earth Ce and contributes to the balanced utilization of rare earth resources.

## 2. Calculation Models and Methods

**Figure 1:**
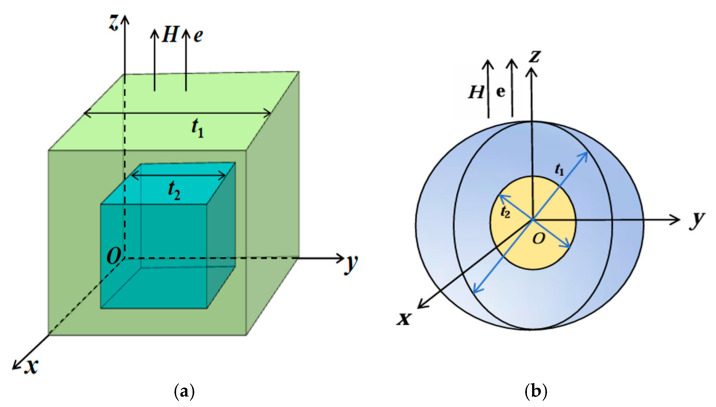
(**Color online**): Computational model of a Nd_2_Fe_14_B/Ce_2_Fe_14_B or Ce_2_Fe_14_B/Nd_2_Fe_14_B structure magnet with an easy axis perpendicular orientation. Here, the variables *t*_1_ and *t*_2_ denote the edge lengths (**a**) (for cubes) or diameters (**b**) (for spheres) of the inner and outer structures, respectively. ((**a**) outer cube: green; inner cube: cyan; (**b**) outer sphere: blue; inner sphere: yellow).

In this study, the MuMax3 micromagnetic software is used to simulate and design structures of Nd_2_Fe_14_B/Ce_2_Fe_14_B and Ce_2_Fe_14_B/Nd_2_Fe_14_B magnets with the easy axis oriented perpendicular to the plane (as shown in Figure 1). The inner and outer cubes share the same body center. A coordinate system O-*xyz* is established with the lower-left vertex of the cube as the origin O, where the *z*-axis is vertically upward, and the plane *z* = 0 corresponds to the *x-y* plane. The easy axis direction ***e*** and the applied magnetic field ***H*** are both aligned along the *z*-axis. In Figure 1a,b, the variables *t_1_* and *t_2_* represent the edge lengths or diameters of the inner and outer cubes or spheres, respectively.

In this paper, the hysteresis loops and energy products of Nd-Ce-Fe-B magnets are calculated. To investigate the effects of the main phase distribution and material thickness on the magnetic properties of Nd-Ce-Fe-B structured permanent magnets, the model assumes a volume ratio of 1:1 between the inner and outer cube phases. The intrinsic parameters of Ce_2_Fe_14_B, namely the exchange energy constant *A* and the volume anisotropy constant *K*, are selected based on the following considerations. Under the assumption of identical crystal structure or point group symmetry, the ratio of *A* between the two magnets is approximately equal to the ratio of their Curie temperatures [22]. Therefore, the value of *A* for Ce_2_Fe_14_B is estimated based on the known *A* of Nd_2_Fe_14_B [22]. *K* of Ce_2_Fe_14_B is then calculated using its known anisotropy field *H_a_* [22], through the relation *K* = *μ*_0_ *M_S_ H_a_*/2. In this study, Nd_2_Fe_14_B and Ce_2_Fe_14_B are selected as the two primary phases of the Nd-Ce-Fe-B magnets, and their intrinsic parameters are listed in Table 1.

The three-dimensional micromagnetic simulations in this work are performed using MuMax3 [26], an open-source software based on the Landau–Lifshitz–Gilbert (LLG) equation [26,27,28,29,30]:

(1) $$\frac{dM}{dt}=-\left|\gamma \right|M\times H_{eff}+\frac{\alpha }{M_{S}}\left(M\times \frac{dM}{dt}\right)$$

where ***M***, ***H****_eff_*, and *γ* represent the magnetization, the effective field, and the gyromagnetic ratio, respectively, with *γ* set to its default value of 2.211 × 10^5^. *M_S_* denotes the saturation magnetization, and *α* is the dimensionless damping coefficient. Although the experimental value of *α* typically ranges from 0.01 to 0.1, it does not affect the equilibrium state of the system. Therefore, a higher value of *α* = 0.5 is used in the simulations to accelerate convergence and reduce computational cost. The effective field is defined as follows:

(2) $$H_{eff}={{-\mu }_{0}}^{-1}\frac{\partial E}{\partial M}.$$

According to the Brown equation [3,8,27], the average energy density *E* is a function of the magnetization ***M***:

(3) $$E=A\left(r\right){\left[\frac{\nabla M}{M_{S}}\right]}^{2}-K\left(r\right)\frac{{\left(M\cdot n\right)}^{2}}{{M_{S}}^{2}}-\mu_{0}M\cdot H-\frac{1}{2}\mu_{0}H_{d}\left(r\right)\cdot M,$$

where ***H*** and ***H****_d_* (***r***) represent the external magnetic field and the demagnetizing field, respectively, and ***n*** is the unit vector along the easy axis direction ***e***. *A* and *K* denote the exchange energy constant and the volume anisotropy constant, respectively. The four terms at the right side of Equation (3) are, in turn, the exchange, anisotropy, applied field (Zeeman), and magnetostatic (demagnetization) energies.

To investigate the effects of main phase distribution and magnet thickness on the magnetic properties of Nd-Ce-Fe-B structured permanent magnets, two models, Nd_2_Fe_14_B/Ce_2_Fe_14_B and Ce_2_Fe_14_B/Nd_2_Fe_14_B, are constructed using the micromagnetic simulation software MuMax3. The side lengths of the inner and outer cubes were set to 8 nm/10 nm, 24.5 nm/30.5 nm, 214 nm/272 nm, 240 nm/304 nm, 324 nm/408 nm, 336 nm/424 nm, and 350 nm/440 nm, respectively. For convenience, the corresponding model indices are summarized in Table 2. The diameters of the inner and outer spheres for the Nd-Ce-Fe-B magnets in the spherical model are 12 nm/15 nm, 30 nm/38 nm, 59 nm/74 nm, 79 nm/100 nm, 398 nm/500 nm, 400 nm/504 nm, and 406 nm/512 nm, respectively. The diameters of the inner and outer spheres for the Nd-Dy-Fe-B magnets in the spherical model are 12 nm/15 nm, 59 nm/74 nm, 398 nm/500 nm, and 406 nm/512 nm, respectively. To ensure that surface and interface anisotropies can be reasonably neglected, it is important that the particle dimensions are much larger than the Bloch domain wall width *δ*, so that the surface-to-volume ratio is low. At the same time, the mesh size was chosen to be smaller than *δ* to guarantee sufficient numerical resolution of domain wall structures and local spin rotations. Accordingly, the cubic model with an edge length of ≤30.5 nm and the spherical model with a diameter of ≤100 nm were discretized using a mesh size of 0.5 nm × 0.5 nm × 0.5 nm, whereas the other models were discretized using a mesh size of 2 nm × 2 nm × 2 nm. The Bloch domain wall widths *δ* of the three materials are 4.2 nm, 6.78 nm, and 4.29 nm for Nd_2_Fe_14_B, Ce_2_Fe_14_B, and Dy_2_Fe_14_B, respectively, as given by the following expression: $\delta =\pi \sqrt{A/K}$. The exchange coupling constant at the interface was taken as the arithmetic mean of the exchange energy constants of the two main phases, Nd_2_Fe_14_B and Ce_2_Fe_14_B, and of Nd_2_Fe_14_B and Dy_2_Fe_14_B, resulting in values of 6.65 × 10^−12^ J·m^−1^ and 7.80 × 10^−12^ J·m^−1^, respectively.

## 3. Results and Discussions

### 3.1. Hysteresis Loops and Magnetic Energy Product

From the demagnetization curves, key physical parameters that characterize the magnetic performance of permanent magnets, including the coercivity *H_C_*, maximum energy product (*BH*)_max_, and remanence *M*_r_, can be obtained. Before describing the nucleation field *H_N_*, pinning field *H_P_*, and coercivity *H_C_*, which can be obtained from the demagnetization curves, we first define these three physical quantities. In the hysteresis loop, the applied external magnetic field brings the magnet to a state of positive saturation, where all magnetic moments are aligned with the external field and the angle *θ* between them is 0°. As the external field decreases, the field value at which the first magnetic moments start to deviate corresponds to the nucleation field (*H_N_* = −*H*), marking the formation of magnetic domains and the onset of the reversible demagnetization process. With the further reduction in the external field, the angle *θ* between the magnetic moments and the applied field gradually increases. When the external field is reduced to the point where all magnetic moments are about to completely reverse, the corresponding negative field value is defined as the pinning field (*H_P_* = −*H*), after which the reversible demagnetization process ends. After pinning, *θ* reaches 180°, and all magnetic moments point downward along the negative *z*-axis, indicating that the magnet has reached negative saturation and all domains have disappeared. The coercivity *H_C_* is defined as the negative value of the external field at which the magnetization of the magnet becomes zero.

**Figure 2:**
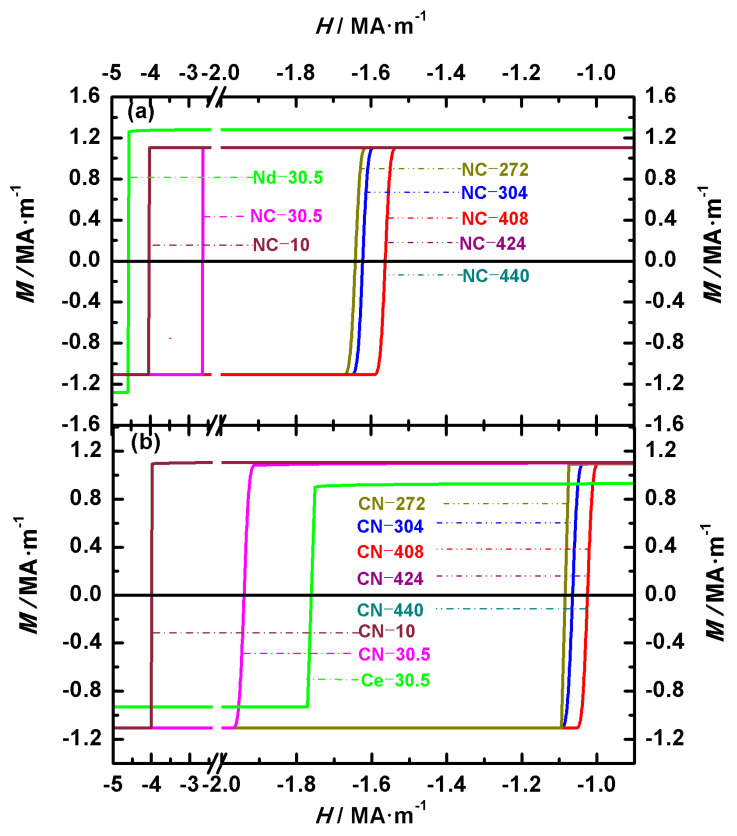
(**Color online**): Demagnetization curves of Nd_2_Fe_14_B/Ce_2_Fe_14_B (**a**) and Ce_2_Fe_14_B/Nd_2_Fe_14_B (**b**) structure magnets with an easy axis perpendicular orientation. For comparison, the hysteresis loops of single-phase Nd_2_Fe_14_B and Ce_2_Fe_14_B cubic magnets (edge length: 30.5 nm), denoted as Nd-30.5 and Ce-30.5, are also shown in Figure 2a,b.

Figure 2 shows the hysteresis loops of Nd-Ce-Fe-B magnets, illustrating the effects of the volume and main phase distribution on their magnetic properties. Figure 2a,b show the loops of Nd_2_Fe_14_B/Ce_2_Fe_14_B (NC) and Ce_2_Fe_14_B/Nd_2_Fe_14_B (CN) magnets, respectively. To compare the differences between biphasic Nd-Ce-Fe-B magnets and single-phase Nd_2_Fe_14_B or Ce_2_Fe_14_B magnets under identical volume and shape conditions, the hysteresis loops of two single-phase cubic magnets with an edge length of 30.5 nm are also shown in Figure 2. These two single-phase magnets are denoted as Nd-30.5 and Ce-30.5, respectively. The two configurations, Nd_2_Fe_14_B/Ce_2_Fe_14_B (abbreviated NC) and Ce_2_Fe_14_B/Nd_2_Fe_14_B (abbreviated CN), have the volume ratio of the two main phases Nd_2_Fe_14_B and Ce_2_Fe_14_B as 1:1. The pinning field, coercivity, and remanence of the NC and CN magnets obtained from the demagnetization curves are summarized in Table 3. In the cubic structure model established in this paper, the volume of the magnet differs based on the inner and outer cubic edge lengths. Thus, the difference in volume is reflected in the variation in the inner and outer cubic edge lengths of the structure in Section 3. As indicated by the calculated magnet numbers in Table 2, the magnets with inner and outer cubic edge lengths of 8 nm/10 nm, 24.5 nm/30.5 nm, 214 nm/272 nm, 240 nm/304 nm, 324 nm/408 nm, 336 nm/424 nm, and 350 nm/440 nm are numbered 10, 30.5, 272, 304, 408, 424, and 440, respectively. As shown in Table 3, for magnets with the same volume and structure, the order of these magnetic properties from highest to lowest is Nd_2_Fe_14_B, Nd_2_Fe_14_B/Ce_2_Fe_14_B, Ce_2_Fe_14_B/Nd_2_Fe_14_B, and Ce_2_Fe_14_B. For the seven Nd_2_Fe_14_B/Ce_2_Fe_14_B magnets, the remanence values for NC-10, NC-30.5, and NC-272 are 1.10586 MA·m^−1^, 1.10570 MA·m^−1^, and 1.10564 MA·m^−1^, respectively, while the remaining four magnets all exhibit a remanence of 1.10563 MA·m^−1^. For the seven Ce_2_Fe_14_B/Nd_2_Fe_14_B magnets, the remanences of CN-10, CN-30.5, CN-272, CN-304, CN-408, CN-424, and CN-440 are 1.10542 MA·m^−1^, 1.10412 MA·m^−1^, 1.10294 MA·m^−1^, 1.10294 MA·m^−1^, 1.10293 MA·m^−1^, 1.10293 MA·m^−1^, and 1.10292 MA·m^−1^, respectively. The remanence values of all magnets remain almost unchanged. This is because the magnetic moments deviate only slightly from the external field prior to complete magnetization reversal. This can also be observed from the hysteresis loops in Figure 2. Comparing the remanence of Nd_2_Fe_14_B/Ce_2_Fe_14_B and Ce_2_Fe_14_B/Nd_2_Fe_14_B, magnets with the same volume but different main phase positions, shows that Nd_2_Fe_14_B/Ce_2_Fe_14_B has a larger remanence. Before the complete magnetization reversal, the moments in Ce_2_Fe_14_B/Nd_2_Fe_14_B are more easily influenced by the demagnetizing field than those in Nd_2_Fe_14_B/Ce_2_Fe_14_B, resulting in a greater deviation from the external field. This behavior, consistent with the subsequent angular distribution analysis, arises from the larger crystalline anisotropy constant (*K*) of Nd_2_Fe_14_B compared to Ce_2_Fe_14_B.

**Figure 3:**
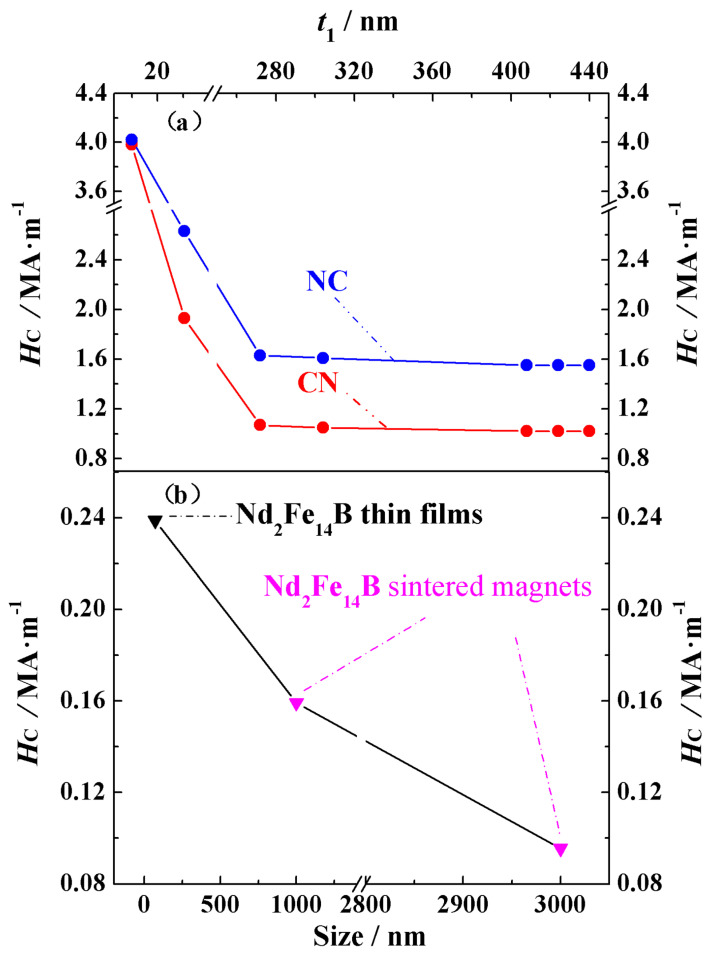
(**Color online**): Comparison of theoretical and experimental coercivity data for (**a**) easy axis perpendicular Nd_2_Fe_14_B/Ce_2_Fe_14_B and Ce_2_Fe_14_B/Nd_2_Fe_14_B magnets and (**b**) Nd_2_Fe_14_B thin films with the c-axis perpendicular to the film plane and sintered magnets [31]. The theoretical calculation data in the figure (**a**) use the horizontal axis symbol *t*_1_ to represent the outer cubic edge length, while the experimental data in (**b**) use the horizontal axis label *Size* to indicate the grain size in the experiments.

The coercivity *H_C_* and pinning field *H_P_* data obtained from the demagnetization curves are summarized in Table 3. Based on the definitions of the pinning field and coercivity given above, it can be seen from the hysteresis loops that the coercivity is equal to the pinning field. Since *H_C_* is equal to *H_P_*, we have placed these two physical quantities in the same column. The coercivity mechanism for all the magnets is pinning. For the Nd_2_Fe_14_B/Ce_2_Fe_14_B structural materials, the coercivity of NC-10, NC-30.5, NC-272, and NC-304 is 4.02 MA·m^−1^, 2.67 MA·m^−1^, 1.63 MA·m^−1^, and 1.61 MA·m^−1^, respectively, while the coercivity of the other three magnets, NC-408, NC-424, and NC-440, is 1.55 MA·m^−1^. For the Ce_2_Fe_14_B/Nd_2_Fe_14_B structural materials, the coercivity of CN-10, CN-30.5, and CN-272, CN-304 is 3.98 MA·m^−1^, 1.93 MA·m^−1^, 1.07 MA·m^−1^, and 1.05 MA·m^−1^, while the coercivity of the other three magnets, CN-408, CN-424, and CN-440, is 1.02 MA·m^−1^. When comparing materials with the same main phase volume but different structures, it is clear that the coercivity of the Nd_2_Fe_14_B/Ce_2_Fe_14_B structure is significantly higher than that of the Ce_2_Fe_14_B/Nd_2_Fe_14_B structure. This is because, from the analysis of the magnetic moment deviation, we found that for Nd-Ce-Fe-B magnets of either structure, the magnetic moments at the outer surface of the cube always start to deviate first. Since Nd_2_Fe_14_B has a larger crystalline anisotropy constant than Ce_2_Fe_14_B, the high-anisotropy moments of Nd_2_Fe_14_B initially resist the applied reverse field, effectively “pinning” the inner cube. As a result, the reversal of the entire magnet requires a larger field, leading to a higher coercivity for the Nd_2_Fe_14_B/Ce_2_Fe_14_B structure compared to the Ce_2_Fe_14_B/Nd_2_Fe_14_B structure. To more clearly compare the changes in coercivity for the fourteen materials and the qualitative trend of the coercivity variation with the grain size observed in the theory and experiment, Figure 3a,b show the theoretical and experimental coercivity data, respectively, as a function of the grain size. From Figure 3a and Table 3, it can be seen that as the material volume increases, the coercivity of both structural types first decreases and then stabilizes. For the two types of structures, the coercivity is almost identical at small volumes. This is because the exchange coupling at the phase interface is stronger in smaller volumes, which even prevents Ce_2_Fe_14_B, when located at the magnet’s surface, from being easily affected by the demagnetizing field despite its relatively small crystalline anisotropy constant. In the experimentally prepared permanent magnets, it has also been observed that coercivity decreases with increasing grain sizes within a certain range, as seen in Nd_2_Fe_14_B thin films with the c-axis perpendicular to the film plane and sintered magnets [31], as well as in SmCo_5_ nanocrystalline materials [32]. As shown in Figure 3b, experimental data indicate that the coercivity of Nd_2_Fe_14_B thin films with the c-axis perpendicular to the film plane and sintered magnets also decreases with increasing grain sizes. Similarly to the simulation results, coercivity decreases more rapidly in materials with smaller grains than in those with larger grains. The coercivity of Nd_2_Fe_14_B thin films with a grain size of 70 nm is 0.23885 MA·m^−1^, while that of Nd_2_Fe_14_B sintered magnets with grain sizes of 1000 nm and 3000 nm is 0.15924 MA·m^−1^ and 0.09554 MA·m^−1^, respectively. This qualitative agreement between theoretical and experimental results confirms the reliability of our theoretical model and indicates that the theoretical calculations provide guidance for both experimental research and industrial production.

**Figure 4:**
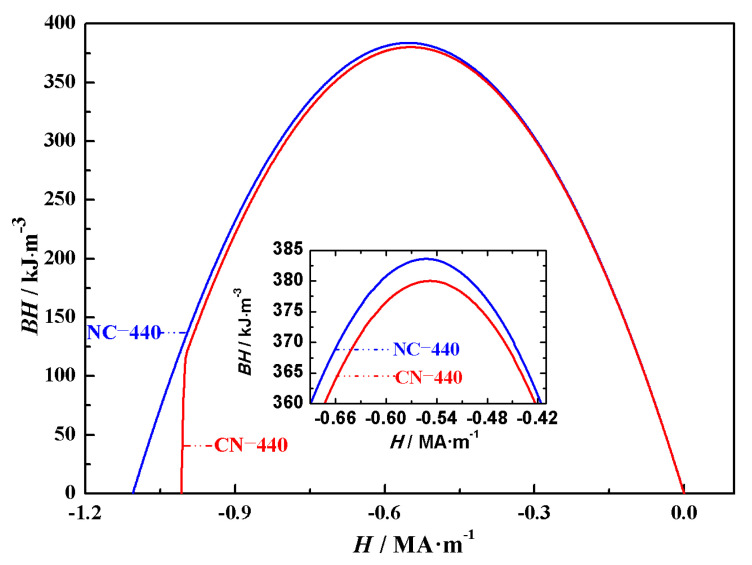
(**Color online**): The energy product (*BH*) of Nd_2_Fe_14_B/Ce_2_Fe_14_B and Ce_2_Fe_14_B/Nd_2_Fe_14_B magnets with inner and outer cubic edge lengths of 350 nm and 440 nm, respectively. The inset provides a magnified view near (*BH*)_max_.

The energy product reflects the ability of a magnet to store and release magnetic energy and is an important parameter for evaluating the performance of permanent magnets. Based on the data obtained from the hysteresis loops in Figure 2, the calculated maximum energy products for the Nd_2_Fe_14_B/Ce_2_Fe_14_B (NC) and Ce_2_Fe_14_B/Nd_2_Fe_14_B (CN) magnets are listed in Table 3. The maximum energy products for NC-10, NC-30.5, NC-272, NC-304, NC-408, NC-424, and NC-440 are 383.95 kJ·m^−3^, 383.80 kJ·m^−3^, 383.72 kJ·m^−3^, 383.71 kJ·m^−3^, 383.69 kJ·m^−3^, 383.70 kJ·m^−3^, and 383.70 kJ·m^−3^, respectively. For CN-10, CN-30.5, CN-272, CN-304, CN-408, CN-424, and CN-440 the maximum energy products are 383.57 kJ·m^−3^, 382.06 kJ·m^−3^, 380.08 kJ·m^−3^, 380.08 kJ·m^−3^, 380.05 kJ·m^−3^, 380.04 kJ·m^−3^, and 380.04 kJ·m^−3^, respectively. The external magnetic field at which the maximum energy product occurs for all fourteen is the same, at -0.56 MA·m^−1^. Furthermore, as explained earlier for the remanence changes in magnets with the same structure, the reason for the similar maximum energy products despite the trend of decreasing values with increasing magnet volumes is that, before the magnetic moments are fully reversed, the deviation of the magnetic moments from the external magnetic field is similar across these magnets. In cases where the main phase distribution is different but the volume is the same, the energy product of Nd_2_Fe_14_B/Ce_2_Fe_14_B magnets is higher than that of Ce_2_Fe_14_B/Nd_2_Fe_14_B magnets. This is because the magnetic moments of Nd_2_Fe_14_B/Ce_2_Fe_14_B magnets are less affected by the external magnetic field compared to Ce_2_Fe_14_B/Nd_2_Fe_14_B magnets, leading to higher magnetization in the former under the same magnetic field. Since the energy product of Nd-Ce-Fe-B magnets shows a similar trend with the external magnetic field, the impact of the main phase positioning on the energy product is analyzed using NC-440 and CN-440, which have inner and outer cubic edge lengths of 350 nm and 440 nm, respectively. As shown in Figure 4, the inset is a magnified view of the energy product curve near its peak. From Figure 4, it can be seen that when the overall volume is the same but the main phase distribution differs, the energy products of the two structures are generally similar, but the inset shows a clear difference in their maximum energy products.

### 3.2. Angular Distribution and In-Plane Magnetization

**Figure 5:**
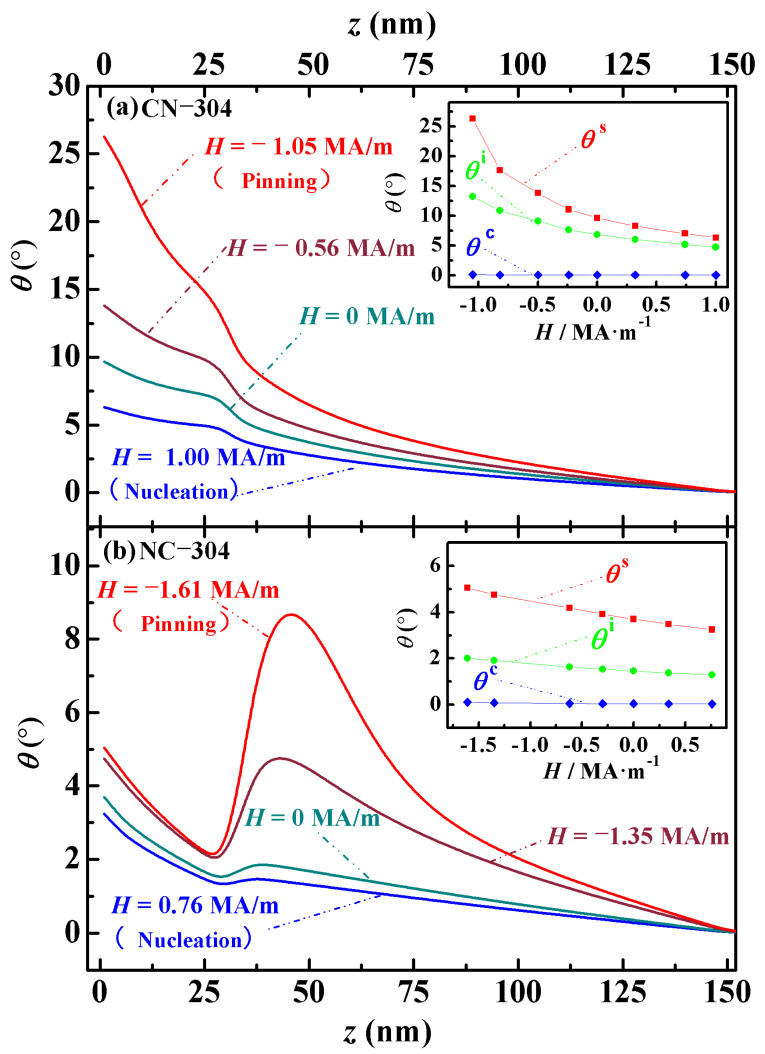
(**Color online**): Angular distributions of two magnets with inner and outer cubic edge lengths of 240 nm and 304 nm, respectively: (**a**) Ce_2_Fe_14_B/Nd_2_Fe_14_B and (**b**) Nd_2_Fe_14_B/Ce_2_Fe_14_B.

The angular distribution *θ*(*z*) clearly describes the deflection behavior of magnetic moments under an external magnetic field and reflects the generation, movement, and evolution of magnetic domains. By analyzing the domain behavior, the magnetic properties of the material can be further evaluated. The angular distribution *θ*(*z*) refers to the variation in the angle *θ* between the magnetic moment and the applied field *H* along the *z*-axis direction. *θ*(*z*) represents the average angle of the magnetic moments within a mesh cell in the *x*–*y* plane at a specific *z*-position. Since the angular distribution of the easy axis perpendicular Nd-Ce-Fe-B magnets exhibits similar trends when the main phase distribution is the same but the volume differs, this study selects two representative structures with inner and outer cubic edge lengths of 240 nm and 304 nm for analysis, as shown in Figure 5. The inset in the upper right corner of Figure 5 illustrates the evolution of three characteristic angles (*θ*^s^, *θ*^i^, and *θ*^c^) with applied magnetic fields. These angles correspond to *θ*(*z*) at the surface of the magnet, the interfaces between the main phases, and the center of the cube along the *z*-direction, respectively. These characteristic *θ*(*z*) values form microscopic hysteresis loops as the external field changes, revealing the intrinsic reversible demagnetization behavior of the magnets. As shown in the model in Figure 1, the magnet structures are designed with a body-centered symmetry, and the angular distributions of all magnets are symmetric around the center of the cube. For simplicity, the analysis of *θ*(*z*) employs only the symmetric lower half of the cube (specifically with *z* ranging from 0 nm to 152 nm). In Figure 5, the angular distribution of the Nd-Ce-Fe-B magnets from left to right corresponds to a bottom-to-top view in the model, shown in Figure 1. In cases with the same total volume but different phase stacking, the Ce_2_Fe_14_B/Nd_2_Fe_14_B magnet undergoes the magnetic moment rotation earlier than the Nd_2_Fe_14_B/Ce_2_Fe_14_B magnet, indicating a lower nucleation field. From nucleation to pinning, the rotation angle *θ*(*z*) at the surface of the Ce_2_Fe_14_B/Nd_2_Fe_14_B magnet is larger than that of the other configuration. This is attributed to the higher crystalline anisotropy constant of Nd_2_Fe_14_B compared to Ce_2_Fe_14_B. During the nucleation-to-pinning process, the Ce_2_Fe_14_B/Nd_2_Fe_14_B and Nd_2_Fe_14_B/Ce_2_Fe_14_B magnets consistently maintain two and six domains, respectively. To further investigate, we also calculated the angular distributions of corresponding Fe/Nd_2_Fe_14_B magnets (replacing Ce_2_Fe_14_B with soft magnetic Fe). The analysis reveals that the domain numbers during nucleation and pinning in Fe/Nd_2_Fe_14_B magnets are identical to those in Ce_2_Fe_14_B/Nd_2_Fe_14_B magnets. As is well known, hard magnetic materials generally exhibit multi-domain states [33]. Thus, based on the above results, although the crystalline anisotropy of Ce_2_Fe_14_B is higher than that of Fe but lower than that of Nd_2_Fe_14_B, the Nd_2_Fe_14_B/Ce_2_Fe_14_B magnets still exhibit characteristics of a hard magnet. In contrast, the Ce_2_Fe_14_B/Nd_2_Fe_14_B magnets with the same total volume do not show the same hard magnetic behavior. This conclusion provides theoretical guidance for the design of high-performance permanent magnet structures.

Taking magnets with inner and outer cubic edge lengths of 240 nm and 304 nm as examples, the domain evolution of both structures is analyzed. Figure 5a shows the symmetric half of the Ce_2_Fe_14_B/Nd_2_Fe_14_B magnet (CN-304), consisting of Ce_2_Fe_14_B (152 nm) and Nd_2_Fe_14_B (120 nm). At the nucleation point (*H =* −*H_N_* = 1.00 MA/m), magnetic domains begin to form, with an initial domain size of 6.24°, defined as the angle difference between two adjacent turning points in the *θ*-*H* curve. As the reverse field increases, the domains continue to grow, reaching 9.60° at *H* = 0 MA/m and further expanding to 13.71° at *H* = −0.56 MA/m. When the reverse field reaches the pinning field (*H =* −*H_P_* = −1.05 MA/m), the domain size increases to 26.16°. After this point, with the continued increase in the reverse field, the magnetic moments flip completely, reaching an angle of 180° with the external field. At this stage, the magnet reaches negative saturation, and the domains vanish. As shown in Figure 5b, the NC-304 magnet begins to nucleate at *H_N_* = −0.76 MA/m. At this point, three domains form with initial sizes of 1.96°, 0.20°, and 1.44°, respectively. As the reverse field increases to *H* = 0 MA/m, these domain sizes grow to 2.25°, 0.41°, and 1.83°. When the field increases to *H* = −1.35 MA/m, the domain sizes become 2.83°, 2.86°, and 4.69°. Finally, at the pinning field (*H =* −*H_P_* = −1.61 MA/m), the domain sizes increase to 3.04°, 6.77°, and 8.67°, respectively. Beyond this point, the further increase in the reverse field causes the magnetic moments to flip fully to 180°, bringing the magnet to negative saturation and eliminating all magnetic domains. From the domain evolution in the NC-304 magnet shown in Figure 5b, it can be seen that after nucleation, with the increasing reverse demagnetizing field, the three domains expand gradually. The central domain grows upward and downward into adjacent regions, increasing its spatial volume. As a result, the adjacent upper and lower domains shrink in spatial volume while still increasing in angular size. As shown in the microscopic hysteresis loop in the inset of Figure 5, for both structures and at all thicknesses, the magnetic moments at the surface of the thin films show a lower resistance to the applied magnetic field. These surface moments influence the magnetic moments at the phase interfaces and inside the central cubic region through exchange interactions. The peaks observed in Figure 5a,b are related to the fact that the magnetocrystalline anisotropy field of Ce_2_Fe_14_B is smaller than that of Nd_2_Fe_14_B. In other words, under the action of an applied reverse magnetic field, the magnetic moments within Ce_2_Fe_14_B are more easily deflected compared to those in Nd_2_Fe_14_B. However, due to the effect of interfacial exchange coupling, the peaks appear near the dual-phase interface (around 32 nm) and also within the primary Ce_2_Fe_14_B phase. That is, during the competition between the different anisotropy fields of the two main phases and the applied reverse field, the deflection of magnetic moments within the magnet gives rise to the peaks shown in the figure. By comparing the angular distributions, it is found that for magnets with the same structure, the deviation angle *θ* of the magnetic moments from the external field is similar between the nucleation and pinning stages.

The number of magnetic domains is determined from the angular distribution. The angular distribution *θ*(*z*) refers to the variation in the angle *θ* between the magnetic moment and the applied field *H* along the z-axis. The horizontal axis represents the position within the magnet, denoted by the variable *z*. Due to the structural symmetry, we only plotted the angular distribution for half of the model volume. Therefore, the number of domains inferred from Figure 5 corresponds to half of the total number of domains in the entire magnet. For a cubic pair magnet with an outer edge length of 304 nm, the range from *z* = 0 nm to 152 nm represents half of its length. The number of magnetic domains is counted by the inflection points in the *θ*(*z*) curve within this range: each inflection point corresponds to one domain. In Figure 5a, no inflection points appear, corresponding to one domain in half of the magnet, so the total number of domains for the Ce_2_Fe_14_B/Nd_2_Fe_14_B structure is two. In Figure 5b, two inflection points appear, corresponding to three domains in half of the magnet, so the total number of domains for the Nd_2_Fe_14_B/Ce_2_Fe_14_B structure is six.

**Figure 6:**
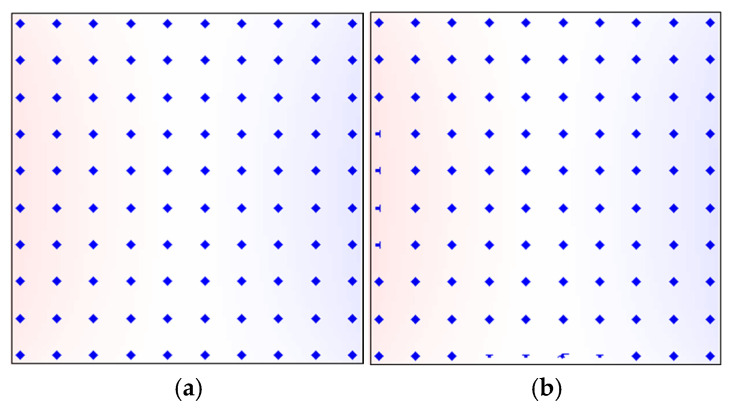

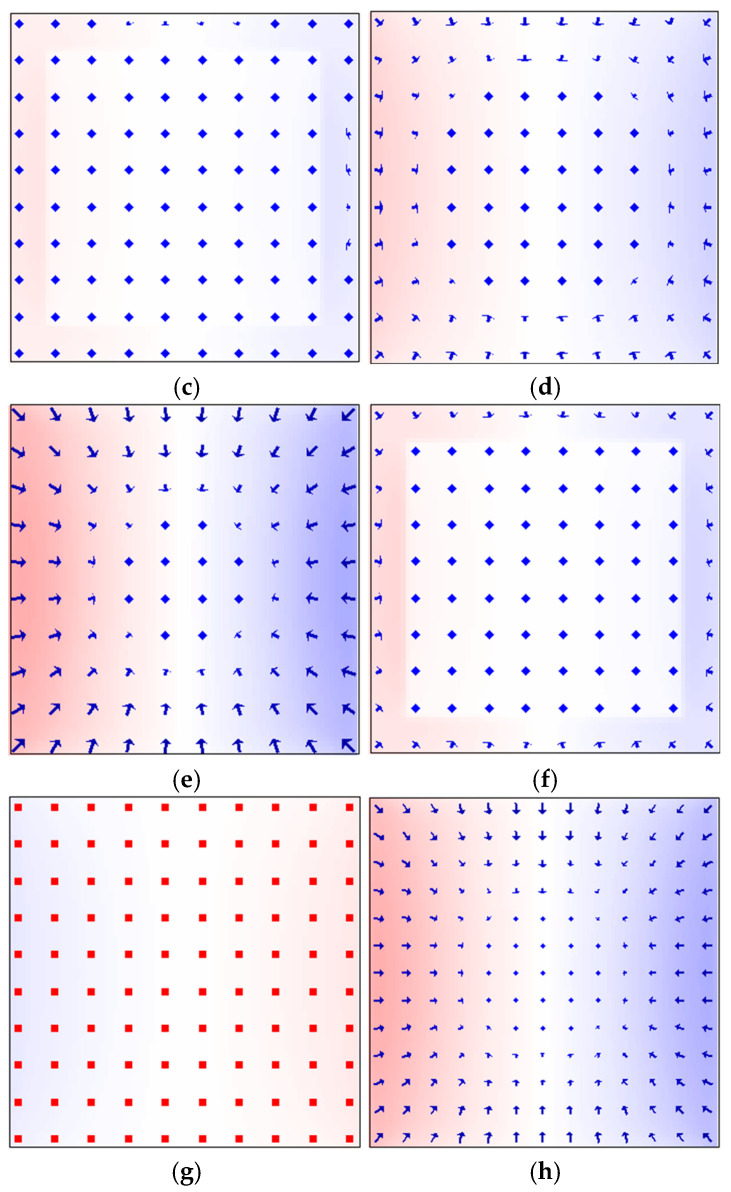
(**Color online**): In-plane magnetic moment distributions of the Ce_2_Fe_14_B (304 nm)/Nd_2_Fe_14_B (240 nm) magnet during the process from nucleation to pinning: (**a**) before nucleation at *H* = 1.07 MA/m, Ce_2_Fe_14_B surface (*z* = 0); (**b**) at nucleation, *H* = −1.00 MA/m, and Ce_2_Fe_14_B surface; (**c**) *H* = −0.42 MA/m, interface layer between Ce_2_Fe_14_B and Nd_2_Fe_14_B; (**d**) *H* = −0.42 MA/m, Ce_2_Fe_14_B surface; (**e**) at pinning, *H* = −1.05 MA/m, Ce_2_Fe_14_B surface; (**f**) at pinning, *H* = −1.05 MA/m, interface layer between Ce_2_Fe_14_B and Nd_2_Fe_14_B; (**g**) after pinning, *H* = −1.07 MA/m, Ce_2_Fe_14_B surface; and (**h**) at pinning, *H* = −1.02 MA/m, Ce_2_Fe_14_B surface of the Ce_2_Fe_14_B (408 nm)/Nd_2_Fe_14_B (324 nm) magnet. The display scale is 1:16, meaning each magnetic moment shown in the figure represents a group of 16 × 16 simulated magnetic moments.

The in-plane magnetization behavior under different external magnetic fields further reveals the magnetization reversal mechanism of permanent magnets. Figure 6 shows the evolution of in-plane magnetic moment distributions in the Ce_2_Fe_14_B (304 nm)/Nd_2_Fe_14_B (240 nm) magnet during the process from nucleation to pinning. All in-plane magnetization views in this study are observed from bottom to top. Figure 6a presents the magnetic moments at the surface of the Ce_2_Fe_14_B (*z* = 0 nm) before the nucleation under *H* = 1.07 MA/m. At this point, the magnetic moments are fully aligned with the external field (*θ* = 0°), along the *z*-axis direction, and the magnet is in a positively saturated state. When the reverse field is reduced to *H* = −*H_N_* = 1.00 MA/m, magnetic moments at two adjacent edges of the Ce_2_Fe_14_B surface begin to deflect first, marking the initiation of magnetic domain formation. This occurs because the vector sum of the demagnetizing field and stray field is maximized at this surface. On the side surfaces of the thin film, no stray field exists due to the easy axis being parallel to those surfaces, so even at saturation, there is no demagnetizing field present. As the reverse field further increases, the surface magnetic moments of Ce_2_Fe_14_B drive the deflection of internal magnetic moments, leading to domain growth (Figure 6b). When the field reaches *H* = −0.42 MA/m, the magnetic moments at the interface layer within the Ce_2_Fe_14_B begin to deflect (Figure 6c). In Figure 6c, the colored area represents the Ce_2_Fe_14_B, while the blank region indicates the interface between Nd_2_Fe_14_B and Ce_2_Fe_14_B. At this point, the surface magnetic moments of Ce_2_Fe_14_B show significantly larger deflections compared to the nucleation stage (Figure 6d). When the reverse field increases to the pinning field (*H* = −*H_P_ =* −1.05 MA/m), the surface magnetic moments of Ce_2_Fe_14_B exhibit their maximum deflection (Figure 6e), corresponding to the largest domain size. Although the surrounding magnetic moments influence the in-plane deflection, the central magnetic moment on the surface of Ce_2_Fe_14_B remains unrotated even at the pinning stage. At the interface, the Ce_2_Fe_14_B moments exhibit further deflection compared to the initial stage, but the Nd_2_Fe_14_B moments remain unchanged (Figure 6f). With the further increase in the reverse field, the system undergoes an irreversible reversal. All magnetic moments are oriented at an angle of *θ* = 180° relative to the external field, reaching the negatively saturated state, with moments pointing downward along the *z*-axis (Figure 6g). These observations confirm the earlier conclusions drawn from the microscopic hysteresis loop analysis: in the Ce_2_Fe_14_B (304 nm)/Nd_2_Fe_14_B (240 nm) magnet, the surface magnetic moments of Ce_2_Fe_14_B respond first to the external field then drive the deflection of interfacial moments through exchange coupling, which in turn induces the reversal of the Nd_2_Fe_14_B moments, completing the demagnetization process. Furthermore, under the same phase distribution, although the pinning field decreases with the increasing magnet volume, the deflection angles at the pinning stage remain nearly the same. This can be observed by comparing the surface magnetic moments of Ce_2_Fe_14_B in the larger-volume Ce_2_Fe_14_B (408 nm)/Nd_2_Fe_14_B (324 nm) magnet (Figure 6h) with those in the Ce_2_Fe_14_B (304 nm)/Nd_2_Fe_14_B (240 nm) magnet (Figure 6e).

### 3.3. Discussion

Considering that the grain morphology in both experiments and industrial production generally lies between cubic and spherical shapes, we further calculated spherical biphasic Nd-Ce-Fe-B magnets. In these models, the two main phases, Nd_2_Fe_14_B and Ce_2_Fe_14_B, have equal volume fractions and are arranged as nested inner and outer spheres, as illustrated in Figure 1b. The configuration with Ce_2_Fe_14_B located in the inner or outer sphere is denoted as Nd_2_Fe_14_B/Ce_2_Fe_14_B (NC) or Ce_2_Fe_14_B/Nd_2_Fe_14_B (CN), respectively. The analysis of the cubic model Nd-Ce-Fe-B magnets shows that the coercivity varies significantly with the spatial position of the main phase, whereas the maximum energy product and other physical quantities change only slightly. Therefore, we focus solely on the trend of the coercivity variation with respect to the main phase volume and spatial position to examine whether it exhibits generality across main phase distributions and different magnet shapes. To ensure that the coercivity results obtained from the cubic model in Figure 1a are also applicable to spherical biphasic models of different shapes and phases, as shown in Figure 1b, we also constructed spherical biphasic Nd-Dy-Fe-B magnets composed of Nd_2_Fe_14_B and Dy_2_Fe_14_B, with phase distributions and volume fractions identical to those of the spherical Nd-Ce-Fe-B magnets; the calculation model is likewise shown in Figure 1b. To facilitate the description of physical parameters for magnets of different sizes, the spherical magnets were labeled in the same manner as the cubic models in Figure 1a and Table 2, using the abbreviation of the rare earth element together with the outer sphere diameter. For example, a pure Dy_2_Fe_14_B magnet is denoted as “Dy” followed by the numerical value of the outer sphere diameter.

**Figure 7:**
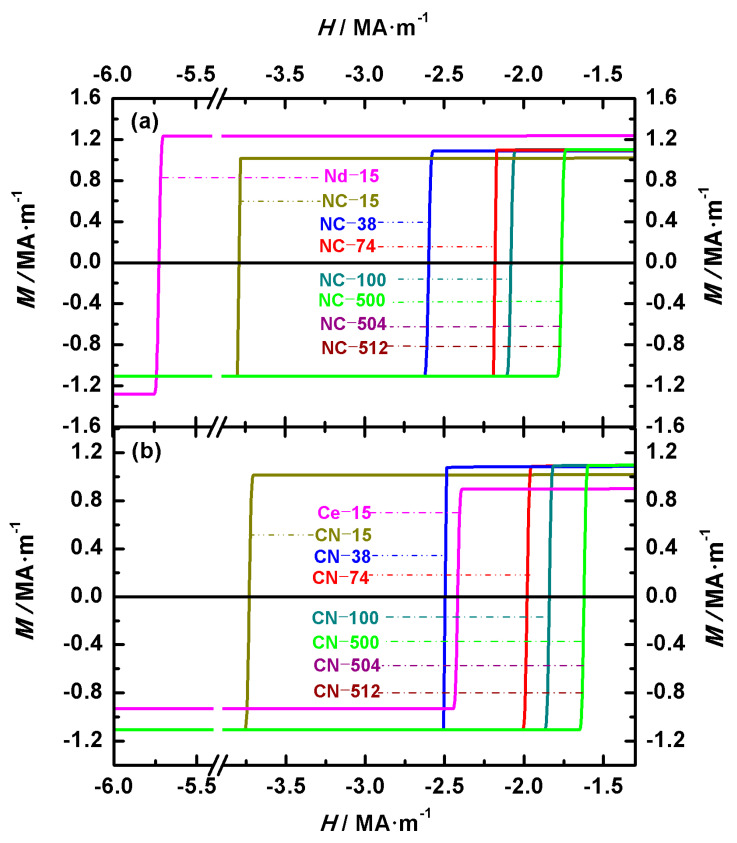
(**Color online**): Hysteresis loops of spherical biphasic Nd-Ce-Fe-B magnets. Two configurations are compared: (**a**) Nd_2_Fe_14_B/Ce_2_Fe_14_B (NC) and (**b**) Ce_2_Fe_14_B/Nd_2_Fe_14_B (CN). For comparison, the hysteresis loops of single-phase Nd_2_Fe_14_B and Ce_2_Fe_14_B spherical magnets with a diameter of 15 nm, denoted as Nd-15 and Ce-15, are also shown in Figure 7a,b.

**Figure 8:**
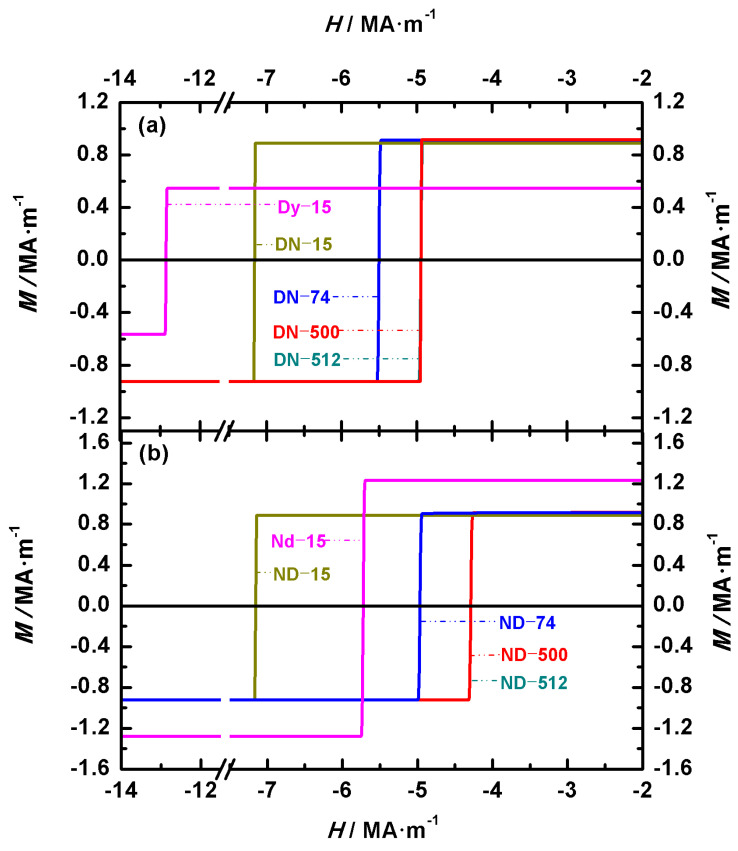
(**Color online**): Hysteresis loops of spherical biphasic Nd-Dy-Fe-B magnets. Two configurations are compared: (**a**) Dy_2_Fe_14_B/Nd_2_Fe_14_B (DN) and (**b**) Nd_2_Fe_14_B/Dy_2_Fe_14_B (ND). For comparison, the hysteresis loops of single-phase Dy_2_Fe_14_B and Nd_2_Fe_14_B spherical magnets with a diameter of 15 nm, denoted as Dy-15 and Nd-15, are also shown in Figure 8a,b.

To analyze the differences in coercivity between biphasic Nd-Ce-Fe-B or Nd-Dy-Fe-B spherical magnets and the corresponding single-phase magnets (Nd_2_Fe_14_B, Ce_2_Fe_14_B, or Dy_2_Fe_14_B), the hysteresis loops of the single-phase systems are also plotted for comparison. The diameters of all three single-phase spheres are 15 nm. Figure 7 and Figure 8 show the hysteresis loops of spherical Nd-Ce-Fe-B and Nd-Dy-Fe-B magnets, respectively. In Figure 7a and 7b, Nd_2_Fe_14_B is located at the outer and inner regions of the biphasic magnets, respectively, whereas in Figure 8a and 8b, Nd_2_Fe_14_B is located at the inner and outer regions of the biphasic magnets, respectively. In the cases where the main phases Nd_2_Fe_14_B, Ce_2_Fe_14_B, and Dy_2_Fe_14_B appear in the biphasic Nd-Ce-Fe-B and Nd-Dy-Fe-B spherical magnets, the hysteresis loops of the corresponding single-phase Nd_2_Fe_14_B, Ce_2_Fe_14_B, and Dy_2_Fe_14_B spheres are also presented. The single-phase models are labeled as Nd-15, Ce-15, and Dy-15, respectively, as shown in Figure 7 and Figure 8. From Figure 7 and Figure 8, the coercivities of single-phase Nd_2_Fe_14_B, Ce_2_Fe_14_B, and Dy_2_Fe_14_B are 5.71 MA·m^−1^, 2.41 MA·m^−1^, and 12.84 MA·m^−1^, respectively. As shown in Table 1, the magnetocrystalline anisotropy fields of single-phase Dy_2_Fe_14_B, Nd_2_Fe_14_B, and Ce_2_Fe_14_B decrease successively. It is well known that the magnetocrystalline anisotropy field determines the coercivity of a magnet. Therefore, when comparing the coercivities of biphasic Nd-Ce-Fe-B and Nd-Dy-Fe-B spherical magnets with those of the corresponding single-phase magnets of the same volume and shape, the coercivity decreases in the following order: Dy_2_Fe_14_B > Dy_2_Fe_14_B/Nd_2_Fe_14_B > Nd_2_Fe_14_B/Dy_2_Fe_14_B > Nd_2_Fe_14_B > Nd_2_Fe_14_B/Ce_2_Fe_14_B > Ce_2_Fe_14_B/Nd_2_Fe_14_B > Ce_2_Fe_14_B.

Figure 7 presents the hysteresis loops of Nd-Ce-Fe-B spherical magnets with outer/inner diameters of 12 nm/15 nm, 30 nm/38 nm, 59 nm/74 nm, 79 nm/100 nm, 398 nm/500 nm, 400 nm/504 nm, and 406 nm/512 nm. Figure 7a,b show the hysteresis loops of the Nd_2_Fe_14_B/Ce_2_Fe_14_B (NC) and Ce_2_Fe_14_B/Nd_2_Fe_14_B (CN) structures, respectively. For the Nd_2_Fe_14_B/Ce_2_Fe_14_B (NC) structure, the coercivities of NC-15, NC-38, NC-74, and NC-100 are 3.78 MA·m^−1^, 2.59 MA·m^−1^, 2.17 MA·m^−1^, and 2.07 MA·m^−1^, respectively, while those of NC-500, NC-504, and NC-512 are all 1.75 MA·m^−1^. For the Ce_2_Fe_14_B/Nd_2_Fe_14_B (CN) structure, the coercivities of CN-15, CN-38, CN-74, and CN-100 are 3.72 MA·m^−1^, 2.49 MA·m^−1^, 1.97 MA·m^−1^, and 1.83 MA·m^−1^, respectively, and those of CN-500, CN-504, and CN-512 are all 1.61 MA·m^−1^. Figure 8 presents the hysteresis loops of Nd-Dy-Fe-B spherical magnets with outer/inner diameters of 12 nm/15 nm, 59 nm/74 nm, 398 nm/500 nm, and 406 nm/512 nm. Figure 8a,b show the hysteresis loops of the Dy_2_Fe_14_B/Nd_2_Fe_14_B (DN) and Nd_2_Fe_14_B/Dy_2_Fe_14_B (ND) structures, respectively. For the Dy_2_Fe_14_B/Nd_2_Fe_14_B (DN) structure, the coercivities of DN-15, DN-74, DN-500, and DN-512 are 7.15 MA·m^−1^, 5.49 MA·m^−1^, 4.94 MA·m^−1^, and 4.94 MA·m^−1^, respectively. For the Nd_2_Fe_14_B/Dy_2_Fe_14_B (ND) structure, the coercivities of ND-15, ND-74, ND-500, and ND-512 are 7.15 MA·m^−1^, 4.96 MA·m^−1^, 4.28 MA·m^−1^, and 4.28 MA·m^−1^, respectively.

**Figure 9:**
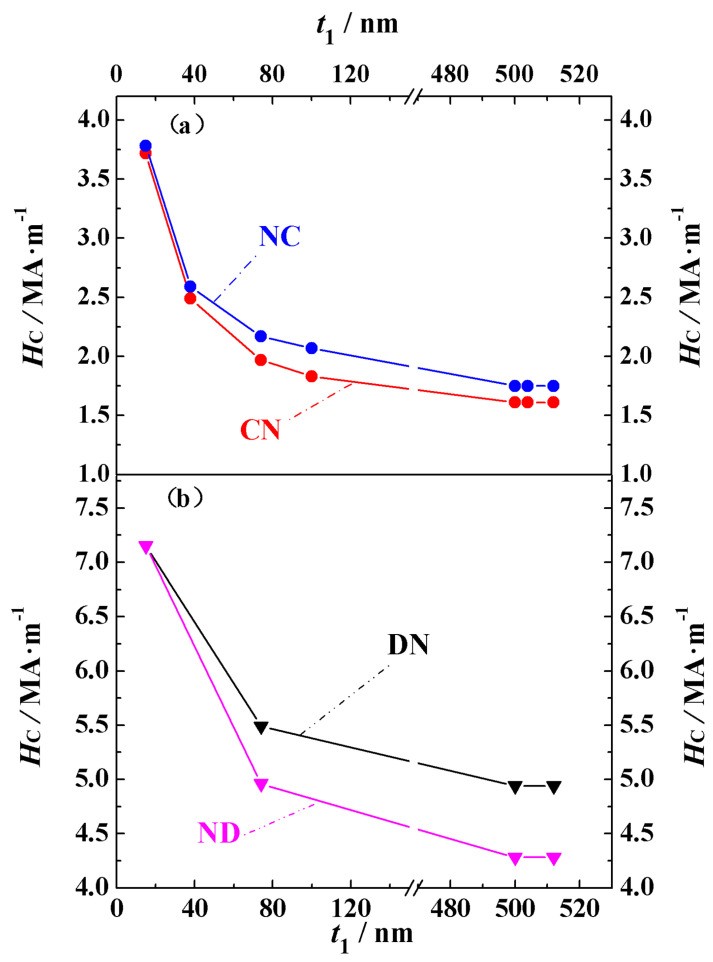
(**Color online**): Coercivity values of biphasic spherical magnets: (**a**) Nd-Ce-Fe-B and (**b**) Nd-Dy-Fe-B. In the figure, NC, CN, DN, and ND represent the Nd_2_Fe_14_B/Ce_2_Fe_14_B, Ce_2_Fe_14_B/Nd_2_Fe_14_B, Dy_2_Fe_14_B/Nd_2_Fe_14_B, and Nd_2_Fe_14_B/Dy_2_Fe_14_B biphasic spherical magnets, respectively. The theoretical calculation data in the figure (**a**,**b**) use the horizontal axis symbol *t*_1_ to represent the outer sphere edge length.

To elucidate the influence of the main phase distribution and volume on the coercivity, the coercivity values of biphasic Nd-Ce-Fe-B and Nd-Dy-Fe-B spherical magnets are presented in Figure 9. Figure 9a,b correspond to the Nd-Ce-Fe-B and Nd-Dy-Fe-B spherical magnets, respectively. As shown in Figure 9, for the spherical biphasic Nd-Ce-Fe-B and Nd-Dy-Fe-B magnets corresponding to the model in Figure 1b, when the main phase with a larger magnetocrystalline anisotropy field surrounds the phase with a smaller anisotropy field, such as Nd_2_Fe_14_B/Ce_2_Fe_14_B (NC) or Dy_2_Fe_14_B/Nd_2_Fe_14_B (DN), the coercivity is higher compared with configurations where the phase positions are reversed, i.e., Ce_2_Fe_14_B/Nd_2_Fe_14_B (CN) or Nd_2_Fe_14_B/Dy_2_Fe_14_B (ND). This difference is small for small volumes but becomes more pronounced as the volume increases. Moreover, when the volume reaches a certain size, the coercivity of all magnets saturates and no longer changes. These qualitative findings are consistent with the conclusions drawn from the cubic model biphasic Nd-Ce-Fe-B magnets in Figure 1a. This conclusion provides theoretical guidance for the structural design of high-coercivity rare earth permanent magnets in both experimental studies and industrial production.

## 4. Conclusions

In this study, either cubic or spherical Nd-Ce-Fe-B structured magnets are designed and simulated using the micromagnetic modeling software MuMax3. These two magnets have the same main phase volume fraction but different spatial distributions of the main phases, namely the Nd_2_Fe_14_B/Ce_2_Fe_14_B structure and the Ce_2_Fe_14_B/Nd_2_Fe_14_B structure. The models are configured with an easy axis perpendicular orientation and varying total volumes to investigate the influence of main phase distributions on magnetic performance. Simulation results show that the coercivity mechanism in both structures is pinning. When the total volume of the magnet is constant, the Nd_2_Fe_14_B/Ce_2_Fe_14_B structure exhibits a significantly higher coercivity and maximum energy product compared to the Ce_2_Fe_14_B/Nd_2_Fe_14_B structure. The coercivity of the magnets shows little variation at small volumes and decreases with increasing volumes, eventually stabilizing beyond a certain threshold, regardless of the phase distribution. This study further demonstrates that, in dual-main magnets with equal phase volumes, positioning the phase with the larger anisotropy field at the particle periphery enhances both the coercivity and the maximum magnetic energy product. This general trend, also observed in Nd-Dy-Fe-B magnets with similar structures, highlights the importance of the phase distribution in optimizing the magnetic performance. The further analysis of the angular distribution reveals that the Nd_2_Fe_14_B/Ce_2_Fe_14_B structure consistently forms six magnetic domains, while the Ce_2_Fe_14_B/Nd_2_Fe_14_B structure only forms two. The in-plane magnetization evolution of the Ce_2_Fe_14_B/Nd_2_Fe_14_B magnet shows that the surface magnetic moments of the Ce_2_Fe_14_B initiate the deflection process during demagnetization. Through exchange coupling, these surface moments gradually drive the rotation of magnetic moments at the interface and in the inner region, completing the overall magnetization reversal. These results highlight the critical role of the phase distribution in optimizing the magnetic performance of Ce-rich Nd-Ce-Fe-B magnets and provide theoretical guidance for the design of high-coercivity, low-cost permanent magnets for industrial applications.

## Figures and Tables

**Table 1:** Intrinsic magnetic properties for two magnetic materials. *A*, *K*, *M_S_*, *H_a_*, and *δ* denote the exchange constant, the volume anisotropy constant, the spontaneous magnetization, magnetocrystalline anisotropy field, and the Bloch domain wall width *δ*, respectively.

Materials.	*A*/J·m^−1^	*K*/J·m^−3^	*M_S_*/A·m^−1^	*H_a_*/MA·m^−1^	*δ*/nm
Nd_2_Fe_14_B [22,23,24,25]	7.70 × 10^−12^	4.30 × 10^6^	1.28 × 10^6^	5.81	4.20
Ce_2_Fe_14_B [22]	5.60 × 10^−12^	1.20 × 10^6^	9.32 × 10^5^	2.07	6.78
Dy_2_Fe_14_B [22]	7.90 × 10^−12^	4.24 × 10^6^	5.65 × 10^5^	11.94	4.29

**Table 2:** Magnet numbers for cube-shaped Nd_2_Fe_14_B/Ce_2_Fe_14_B and Ce_2_Fe_14_B/Nd_2_Fe_14_B magnetic materials.

Table.	Nd_2_Fe_14_B/Ce_2_Fe_14_B	Ce_2_Fe_14_B/Nd_2_Fe_14_B
8 nm/10 nm	NC-10	CN-10
24.5 nm/30.5 nm	NC-30.5	CN-30.5
214 nm/272 nm	NC-272	CN-272
240 nm/304 nm	NC-304	CN-304
324 nm/408 nm	NC-408	CN-408
336 nm/424 nm	NC-424	CN-424
350 nm/440 nm	NC-440	CN-440

**Table 3:** The pinning field *H_P_*, the coercivity *H_C_*, the maximum energy products (*BH*)_max_, and the remanence *M*_r_ of cube-shaped Nd_2_Fe_14_B/Ce_2_Fe_14_B and Ce_2_Fe_14_B/Nd_2_Fe_14_B magnets. To make the mechanism of coercivity clearer and to simplify the table, the coercivity and pinning field data are presented in a single column.

Materials	*H_C_* (*H_P_*)/MA·m^−1^	*M*_r_/MA·m^−1^	(*BH*)_max_/kJ·m^−3^
Ce-30.5	1.75	0.93085	271.65
Nd-30.5	4.56	1.27947	513.91
CN-10	3.98	1.10542	383.57
NC-10	4.02	1.10586	383.95
CN-30.5	1.93	1.10412	382.06
NC-30.5	2.67	1.10570	383.80
CN-272	1.07	1.10294	380.08
NC-272	1.63	1.10564	383.72
CN-304	1.05	1.10294	380.08
NC-304	1.61	1.10563	383.71
CN-408	1.02	1.10293	380.05
NC-408	1.55	1.10563	383.69
CN-424	1.02	1.10293	380.04
NC-424	1.55	1.10563	383.70
CN-440	1.02	1.10292	380.04
NC-440	1.55	1.10563	383.70
